# Awareness of Acute Stroke Among the General Population in the Western Region of Saudi Arabia

**DOI:** 10.7759/cureus.51979

**Published:** 2024-01-09

**Authors:** Shrooq M Hawati, Fares Binobaid, Rawya Zeed Melybari, Samer Alabdali, Ghadi Alhazmi, Alaa Namankani, Haneen A Abdrabuh

**Affiliations:** 1 Emergency, Security Forces Hospital, Makkah, SAU; 2 Emergency Medicine, King Abdulaziz Hospital, Makkah, SAU; 3 Medicine and Surgery, Umm Al-Qura University, Makkah, SAU

**Keywords:** stroke awareness, stroke prevention, stroke symptoms, stroke risk factors, acute stroke, cross sectional studies, stroke knowledge, awareness, awareness of acute stroke

## Abstract

Background

A stroke is an abrupt neurological deficit that occurs due to a vascular origin. Stroke is one of the main causes of functional disability and irreversible brain damage globally. Following cancer and ischemic heart disease, stroke ranks as the third-highest contributor to adult mortality. According to studies conducted in Saudi Arabia, the estimated annual incidence of stroke was 29.8 per 100,000 individuals. Patients who are at risk for stroke and their families should be aware of the danger of stroke and be familiar with the symptoms. Stroke warning signs include abrupt weakness in the face or limbs, confusion, speech slurring, vision problems, difficulty in walking, and severe headache with no obvious explanation. The burden of stroke consequences can be greatly reduced by early detection of stroke symptoms and prompt treatment for acute attacks. A delay in seeking medical attention following a stroke attack has been linked to poor knowledge of stroke symptoms and risk factors. Furthermore, an important factor in reducing the incidence of stroke is public awareness of risk factors, symptoms, and prevention strategies. General awareness of stroke leads to a proper response to stroke, pursuit of medical treatment, and a better outcome.

Aim

The aim of this study is to assess the awareness of acute stroke among the general population in the Western Region of Saudi Arabia.

Methodology

A cross-sectional study was conducted in 2023 in the western region of Saudi Arabia. An online, validated, self-administered questionnaire was distributed randomly. The estimated sample size was 385, and 539 were the collected responses.

Results

More than half of the respondents were females (66.0%) (n=356), aged 20 to 29 years (50.3%) (n=271). In general, the majority of respondents had correctly identified that the elderly are at high risk for stroke (92.8%) (n=500) and that stroke can cause death (81.6%) (n=440). More than half of the population under study (59.9%) (n=323) correctly indicated that stroke is preventable. However, only (11.3%) (n=61) of them stated that the ideal timeframe to initiate thrombolysis in acute cases is 4.5 hours. Regarding the risk factors of stroke, the most frequently identified factors included hypertension (84.4%) (n=455) and heart disease (64.6%) (n=347). When the participants were asked about the warning signs and symptoms of stroke, the most commonly identified response was confusion (70.3%) (n=379). A good knowledge level was prevalent among 311 participants (57.7%, 95% CI, 53.4 to 61.9). Good knowledge levels were significantly higher among participants aged 20 to 29 years (63.5%) (n=172) and 60 years or more (65.2%) (n=15) compared to other categories. A significantly higher proportion of non-Saudis had higher knowledge levels ((75.9%) (n=22) versus (56.7%) (n=289), p = 0.042). Results of the multivariate analysis showed that participants aged 40 to 49 years (OR = 0.54, 95% CI, 0.33-0.89; p = 0.016) and 50 to 59 years (OR = 0.37, 95% CI, 0.21 to 0.63, p < 0.001) were less likely to have good knowledge levels.

Conclusion

The findings of this study indicate that (57%) (n=307) of participants had adequate knowledge of acute stroke. However, public education programs are still required to further expand this knowledge.

## Introduction

A stroke is an abrupt neurological deficit that occurs due to a vascular origin [1]. Stroke is one of the main causes of functional disability and irreversible brain damage globally. According to the World Health Organization (WHO), 15 million individuals worldwide suffer from stroke every year, of which five million pass away and five million remain permanently disabled [2]. Following cancer and ischemic heart disease, stroke ranks as the third-highest contributor to adult mortality. Approximately 65% of deaths due to stroke have been reported in developing countries, with nearly 40% of these stroke deaths occurring in people under the age of 70 [2]. According to studies conducted in Saudi Arabia, the estimated annual incidence of stroke was 29.8 per 100,000 individuals [3]. There are two categories of stroke risk factors: modifiable and non-modifiable. Non-modifiable risk factors include age, genetic disorders, history of transient ischemic attack, and family history of cardiovascular or cerebrovascular disease. Modifiable risk factors include systemic hypertension, hyperlipidemia, diabetes mellitus, atherosclerosis, cardiovascular diseases, sedentary lifestyle, obesity, smoking, alcohol intake, and recreational drug use [4-6]. Patients who are at risk for stroke and their families should be aware of the danger of stroke and be familiar with the symptoms. Stroke warning signs include abrupt weakness in the face or limbs, confusion, speech slurring, vision problems, difficulty in walking, and severe headache with no obvious explanation. The burden of stroke consequences can be greatly reduced by early detection of stroke symptoms and prompt treatment for acute attacks [7]. A delay in seeking medical attention following a stroke attack has been linked to poor knowledge of stroke symptoms and risk factors [8]. Furthermore, an important factor in reducing the incidence of stroke is public awareness of risk factors, symptoms, and prevention strategies [9]. In 2021, research conducted in the Czech Republic with 1,004 participants to evaluate stroke awareness revealed that the overall level of awareness about stroke is high in the Czech Republic [10]. In Egypt, a study done in 2018 revealed that individuals had a poor level of stroke awareness. Significant predictors of greater awareness of stroke include higher wealth levels, better education, the presence of risk factors, and knowing someone who previously had a stroke [11]. In 2021, a study was conducted in Riyadh, Saudi Arabia, to assess the awareness of stroke. The study included 150 participants and found that the majority of them had a high level of awareness regarding the risk factors of stroke (76.66%) [9]. Additionally, over half of the participants (63.9%) showed a moderate level of awareness about the signs and symptoms of stroke [9]. Another study conducted in Abha, Saudi Arabia, revealed a significant lack of knowledge among the population regarding acute stroke warning symptoms, risk factors, and appropriate stroke response [12]. According to a study conducted in Jeddah, the majority of participants recognized stroke (81.46%), and 59.2% correctly identified it [12]. General awareness of stroke leads to a proper response to stroke, pursuing medical treatment, and a better outcome [12]. Due to the limited studies that assess awareness level of stroke in the Western Region of Saudi Arabia, our study aimed to assess the awareness of acute stroke among the general population in the Western Region of Saudi Arabia.

## Materials and methods

Study design

A cross-sectional study was conducted in 2023 in the Western Region of Saudi Arabia to assess the awareness of acute stroke among the general population. An online, self-administered questionnaire was randomly distributed to participants.

Study population

The study included individuals above 18 years of age from the general population in the Western Region of Saudi Arabia. However, individuals working or studying in the medical sector were excluded from the study.

Sampling methodology

Data were collected through an online validated survey using Google Documents. The survey was electronically distributed through social media platforms like WhatsApp, Twitter, and Telegram. The purpose of the study and the voluntary participation were informed to the participants. Inclusion criteria were explained at the beginning of the survey, and no identifier information was collected to maintain privacy and confidentiality. The data were only permitted for access by the investigators. The Raosoft sample size calculation was used to determine the sample size. The estimated sample size was calculated as 385 participants to achieve a precision of 5% with a 95% confidence interval.

Study tool

The questionnaire used in this study aimed to gather information about participants' awareness of acute stroke. It consisted of two sections.

Sociodemographic Data

This section collected information on participants' age, gender, educational level, residency, and previous knowledge about stroke.

Assessment of Awareness of Acute Stroke

This section included questions related to participants' knowledge of stroke risk factors, warning signs and symptoms, preventability of stroke, and sources of information about stroke. Native Arabic speakers translated the questionnaire for the participants.

Validity and reliability analysis

The questionnaire was developed based on existing literature on stroke awareness and reviewed by experts in the field. Questions in the present study covered the knowledge level about stroke. The questionnaire items were derived from previous studies done in Saudi Arabia [6,9]. A pilot study was done that included 25 participants. The results of the pilot study were used to assess reliability and validity.

Questionnaire Validation

A panel of three specialists evaluated the questionnaire's validity. The experts modified the first items to see if they were ready for evaluation. The subject matter experts were tasked with rating each item on a 4-point scale based on its applicability and relevance. The content validity index (CVI) is the percentage of all questionnaire items that received a score of 3 or 4 from experts (Table 1).

**Table 1:** The content validity index (CVI)

Item	Score
Adequate (simple, relevant and clear)	4
Adequate but needs minor revision	3
Needs major modification	2
Not so adequate (can be omitted)	1

A score of 80% is generally considered to have good validity. The calculated CVI of the study's questionnaire was 91%.

Reliability Analysis

A Cronbach's alpha value of 0.83 was revealed when the reliability of the internal consistency of the study questionnaire was tested. A Cronbach's alpha score greater than 0.7 indicates that the scale is deemed internally consistent [13]. The questionnaire is provided in the appendix.

Statistical analysis

Statistical analyses were performed using RStudio (R version 4.1.1). Categorical variables were presented as frequencies and percentages, while numerical variables were expressed as medians and interquartile ranges (IQR). A knowledge score was calculated by summing up the correct responses to knowledge items (n = 15), ranging from 0 to 15. A score equal to or above the median score was considered a good knowledge level. The prevalence of good knowledge was determined using a one-sample proportion test with a 95% confidence interval (CI). Factors associated with a good knowledge level were assessed using Pearson's chi-squared test or Fisher's exact test. Predictors of good knowledge were examined through multivariate binomial logistic regression by incorporating significantly associated factors. The results were presented as odds ratios (OR) with 95% CIs. Statistical significance was set at a p-value of < 0.05.

## Results

Initially, we collected 750 responses. However, we excluded 22 records from participants who were residing outside the Western region, 69 records of those who were working in the medical field, and 120 records of participants aged < 20 years. Therefore, data from 539 participants were ultimately analyzed in the current study.

Sociodemographic characteristics

More than half of the respondents were females (66.0%) (n=356) aged 20 to 29 years (50.3%) (n=271). The majority of respondents were Saudis (94.6%) (n=510), residing in urban regions (95.5%) (n=515), and had received a university degree or higher (79.0%) (n=426). Medina and Mecca residents represented (41.4%) (n=223) and (23.0%) (n=124) of the participants, respectively. A large percentage of the participants had heard about stroke (91.8%) (n=495), and (10.9%) (n=59) of them had experienced a personal or family history of stroke (Table 2).

**Table 2:** Sociodemographic characteristics

Parameter	Category	N	%
Gender	Male	183	34.0
	Female	356	66.0
Age	20 to 29	271	50.3
	30 to 39	67	12.4
	40 to 49	97	18.0
	50 to 59	81	15.0
	60 or more	23	4.3
Nationality	Saudi	510	94.6
	Non-Saudi	29	5.4
Geographical Region	Mecca	124	23.0
	Medina	223	41.4
	Jeddah	98	18.2
	Taif	65	12.1
	Others	29	5.4
Residency	Urban Area	515	95.5
	Rural Area	24	4.5
Educational level	Elementary	5	0.9
	Middle school	14	2.6
	High school	94	17.4
	Higher	426	79.0
Employment	Student	164	30.4
	Unemployed	132	24.5
	Employed	175	32.5
	Entrepreneur	19	3.5
	Retired	49	9.1
Ever heard about stroke	Yes	495	91.8
Personal or family history of stroke	Yes	59	10.9

Participants’ responses to knowledge items

In general, the majority of respondents had correctly identified that the elderly are at high risk for stroke (92.8%) (n=500) and that stroke can cause death (81.6%) (n=440). More than half of the population under study (59.9%) (n=323) correctly indicated that stroke is preventable. However, only 11.3% (n=61) of them stated that the ideal timeframe to initiate thrombolysis in acute cases is 4.5 hours (Table 3).

**Table 3:** Participants’ responses to knowledge items

Parameter	Category	N	%
Who is at high risk for stroke?	Children	4	0.7
	Young	35	6.5
	Elderly	500	92.8
Time frame to initiate thrombolysis in acute ischemic stroke	2 hours	313	58.1
	3 hours	113	21.0
	3.5 hours	52	9.6
	4.5 hours	61	11.3
Stroke can cause death	No	15	2.8
	Yes	440	81.6
	Do not know	84	15.6
Stroke is preventable	No	13	2.4
	Yes	323	59.9
	Do not know	203	37.7

**Figure 1:**
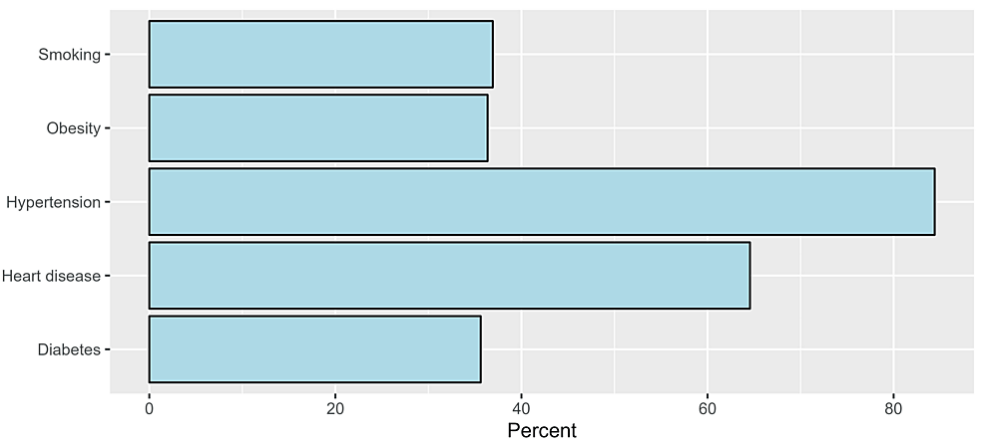
Participants’ responses to the risk factors of stroke.

Regarding the risk factors of stroke, the most frequently identified factors included hypertension (84.4%) (n=455) and heart disease (64.6%) (n=348), whereas the least frequently reported factors were diabetes (35.6%) (n=192) and obesity (36.4%) (n=196) (Figure 1).

**Figure 2:**
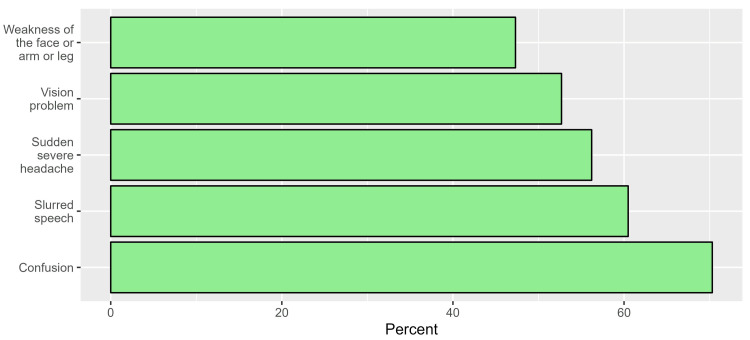
Participants’ responses to the warning signs and symptoms of stroke.

When the participants were asked about the warning signs and symptoms of stroke, the most commonly identified responses were confusion (70.3%) (n=379) and slurred speech (60.5%) (n=326). Conversely, weakness of the face, arms, and legs and vision problems were the least identified warning signs ((47.3%) (n=255) and (52.7%) (n=284)) respectively (Figure 2).

**Figure 3:**
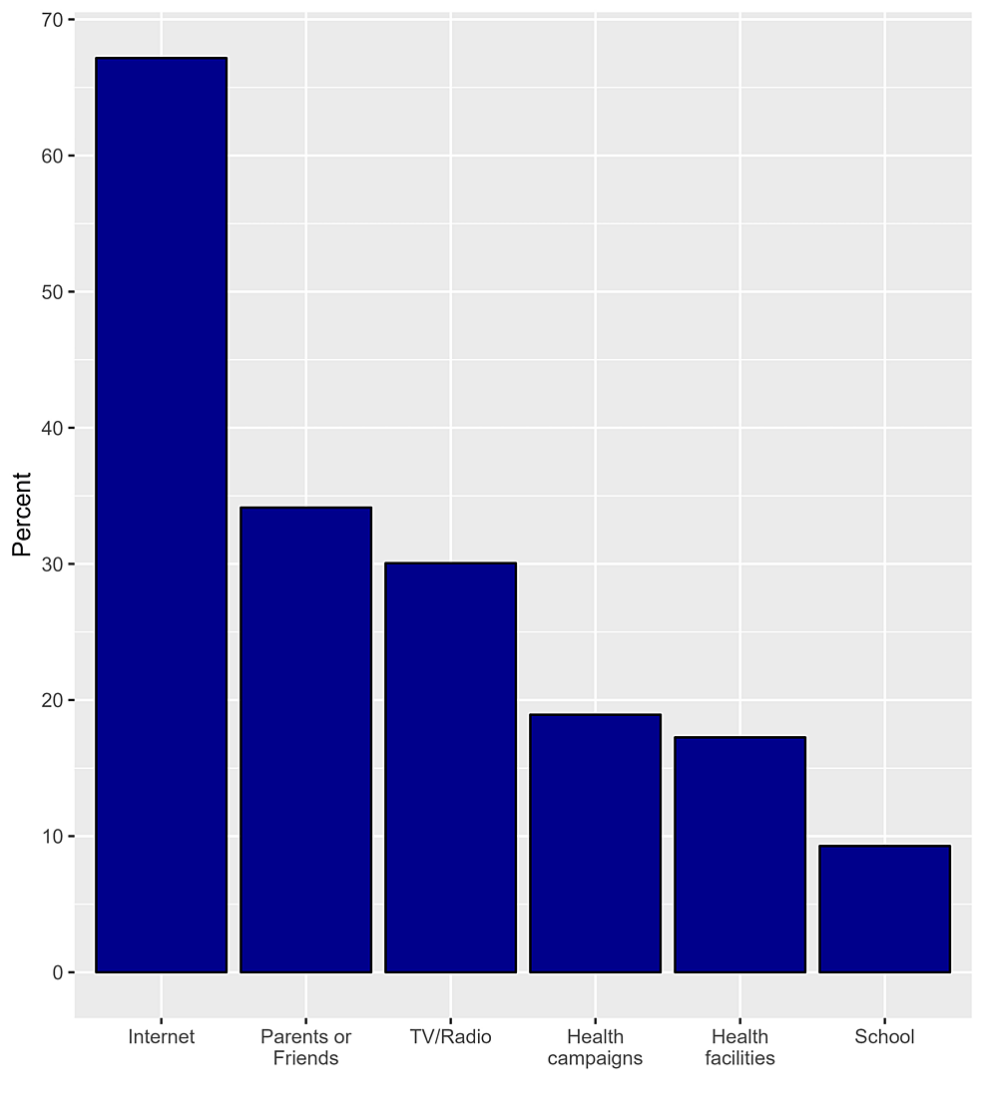
The proportions of sources of information regarding stroke.

Considering the sources of information, participants declared that the most common sources are the internet (67.2%) (n=362), parents or friends (34.1%) (n=184), and TV or radio (30.1%) (n=162). Schools were the least used source of information (9.3%) (n=50) (Figure 3).

Characteristics of the knowledge score

Based on a total of 15 items, the median (IQR) knowledge score was 8.0 (6.0 to 10.0) with a minimum of 2 and a maximum of 15. A good knowledge level was prevalent among 311 participants (57.7%, 95% CI, 53.4 to 61.9).

Factors associated with a good knowledge level

Good knowledge levels were significantly higher among participants aged 20 to 29 years (63.5%) (n=172) and 60 years or more (65.2%) (n=15) compared to other categories, including 30 to 39 years (59.7%) (n=40), 40 to 49 years (52.6%) (n=51), and 50 to 59 years (40.7%, n=33, p = 0.005). A significantly higher proportion of non-Saudis had higher knowledge levels ((75.9%) (n=22) versus (56.7%) (n=289), p = 0.042). Furthermore, participants who had knowledge about stroke had significantly higher knowledge levels than those who did not have much knowledge ((59.6%) (n=295) versus (40.4%) (n=200), p = 0.003). Knowledge about stroke was also significantly higher among participants who obtained their knowledge from TV/radio ((70.4%) (n=114) versus (29.6%) (n=48); p < 0.001), the internet ((63.3%) (n=229) versus (36.7%) (n=133); p < 0.001), school ((76.0%) (n=38) versus (24.0%) (n=12); p = 0.006), health campaigns ((71.6%) (n=73) versus (28.4%) (n=29); p = 0.002), and healthcare facilities ((67.7%) (n=63) versus (32.3%) (n=30); p = 0.031) as shown in Table 4.

**Table 4:** Factors associated with knowledge levels among participants under study.

Parameter	Category	Good knowledge level					p
		No, N = 228		Yes, N = 311			
		N	%		N	%	
Gender	Male	78	42.6		105	57.4	0.914
	Female	150	42.1		206	57.9	
Age	20 to 29	99	36.5		172	63.5	0.005
	30 to 39	27	40.3		40	59.7	
	40 to 49	46	47.4		51	52.6	
	50 to 59	48	59.3		33	40.7	
	60 or more	8	34.8		15	65.2	
Nationality	Saudi	221	43.3		289	56.7	0.042
	Non-Saudi	7	24.1		22	75.9	
Geographical Region	Mecca	47	37.9		77	62.1	0.128
	Medina	94	42.2		129	57.8	
	Jeddah	39	39.8		59	60.2	
	Taif	37	56.9		28	43.1	
	Others	11	37.9		18	62.1	
Residency	Urban Area	220	42.7		295	57.3	0.363
	Rural Area	8	33.3		16	66.7	
Educational level	Elementary	3	60.0		2	40.0	0.750
	Middle school	7	50.0		7	50.0	
	High school	41	43.6		53	56.4	
	Higher	177	41.5		249	58.5	
Employment	Student	59	36.0		105	64.0	0.249
	Unemployed	60	45.5		72	54.5	
	Employed	81	46.3		94	53.7	
	Entrepreneur	6	31.6		13	68.4	
	Retired	22	44.9		27	55.1	
Ever heard about stroke	Yes	200	40.4		295	59.6	0.003
Personal or family history of stroke	Yes	21	35.6		38	64.4	0.269
Sources of information	TV/Radio	48	29.6		114	70.4	<0.001
	Internet	133	36.7		229	63.3	<0.001
	School	12	24.0		38	76.0	0.006
	Parents or Friends	71	38.6		113	61.4	0.209
	Health campaign	29	28.4		73	71.6	0.002
	Health facilities	30	32.3		63	67.7	0.031

Predictors of good knowledge

Results of the multivariate analysis showed that participants aged 40 to 49 years (OR = 0.54, 95% CI, 0.33-0.89; p = 0.016) and 50 to 59 years (OR = 0.37, 95% CI, 0.21 to 0.63, p < 0.001) were less likely to have good knowledge levels. In contrast, good knowledge levels were independently associated with having knowledge about stroke (OR = 2.79, 95% CI 1.41-5.72; p = 0.004) in addition to obtaining knowledge about stroke from TV or radio (OR = 2.19, 95% CI 1.45-3.36; p < 0.001), the internet (OR = 1.93, 95% CI 1.30-2.88; p = 0.001), and health campaigns (OR = 2.03, 95% CI 1.22-3.46; p = 0.008) as shown in Table 5.

**Table 5:** Results of the predictors of good knowledge regarding stroke. OR = Odds Ratio, CI = Confidence Interval, Ref: a reference category

Parameter	Category	OR	95% CI	p
Age	20 to 29	Ref	Ref	
	30 to 39	0.81	0.45, 1.45	0.466
	40 to 49	0.54	0.33, 0.89	0.016
	50 to 59	0.37	0.21, 0.63	<0.001
	60 or more	1.14	0.45, 3.07	0.793
Nationality	Saudi	Ref	Ref	
	Non-Saudi	1.55	0.64, 4.18	0.354
Ever heard about stroke	No	Ref	Ref	
	Yes	2.79	1.41, 5.72	0.004
TV/Radio	No	Ref	Ref	
	Yes	2.19	1.45, 3.36	<0.001
Internet	No	Ref	Ref	
	Yes	1.93	1.30, 2.88	0.001
School	No	Ref	Ref	
	Yes	1.57	0.77, 3.37	0.230
Health campaign	No	Ref	Ref	
	Yes	2.03	1.22, 3.46	0.008
Health facilities	No	Ref	Ref	
	Yes	1.60	0.93, 2.77	0.091

## Discussion

The incidence of stroke is rising globally. In 2016, 5.5 million deaths worldwide due to stroke were reported with 8,539 of those deaths taking place in Saudi Arabia [14]. It has been demonstrated that increasing stroke education and awareness among different populations greatly reduces pre-hospital delay and morbidity [15]. In our research, most of the participants (92.8%) (n=500) declared that the elderly had a higher risk for stroke. In a similar study carried out among students at Jazan University, Old age was identified by 60.4% of participants as a risk factor for stroke [16]. The study's participants stated that they are fully aware that strokes can be prevented and cause death. However, according to a prior study, they are unsure of how to adequately prevent it [17]. In addition, individuals at high risk of stroke do not follow prevention advice even when medical professionals verbally advise them to do so [18]. Individuals understand that emergency medical interventions are immediately required for stroke treatment [19,20], but one interesting finding in the current study is that only 11.3% (n=61) of participants were aware of the exact timeframe of thrombolytic administration. The possible explanation for this result is a lack of adequate focus on this point concerning the timeframe in the different information resources. According to the study's findings, heart disease is the second most common risk factor for stroke, after hypertension. A study conducted in Lebanon with 390 participants indicated that nearly 80% recognized hypertension and psychological stress as the most prevalent risk factors [19]. When it comes to stroke warning signs and symptoms, the most frequently identified responses were confusion (70.3%) (n=379) and slurred speech (60.5%) (n=326). These findings are consistent with some published studies [21-23]. However, they differ from a study by Alhazzani that was conducted in Abha City, Saudi Arabia, which revealed that sudden severe headache was the most frequently identified response (54.1%) while slurred speech and confusion were the third and sixth most frequently identified symptoms (44.6% and 17.9%, respectively) [24]. Most participants used the internet as their primary source of information regarding stroke. In a similar study conducted in Madina City, Saudi Arabia, participants who obtained their knowledge from the internet and social media (65.77% and 35%, respectively) had higher knowledge about stroke than those who obtained their knowledge from other sources [23]. We can utilize the public media on the internet to increase public awareness of stroke, which definitely would positively influence the knowledge of stroke among the Saudi population. This study shows a significant increase in knowledge among participants of younger ages, such as 20 to 29, and those who are 60 or older compared to a similar study done in Riyadh 2021 in which it was found that the older population aged 45 and older acquired the highest levels of knowledge (50%) compared to the other groups [8]. In addition, a significant increase in knowledge among non-Saudi participants was found. This result is similar to a study done in Qassim, Saudi Arabia, in which it was found that non-Saudis had significantly higher levels of knowledge about stroke [25]. Assessing the level of awareness will help people understand the need for public education programs to increase awareness about this critical disease. This increase in knowledge may affect how quickly the patient is treated and will lead to the prevention of permanent damage and complications as the early start of medical treatment and recovery from a stroke attack often depend on the awareness of the family members. We use a validated online survey to assess awareness of acute stroke among our population. In addition, the study was carried out in multiple cities in the Western region of Saudi Arabia; thus, this setting is another strength. The homogeneity of our research sample imposed a limitation on the study and made it challenging to generalize the results to the entire population since 79% (n=426) of our participants obtained a university degree or higher. In addition, since it was an online survey, it is possible that people who are not familiar with social media and the Internet were not included. Increasing the knowledge of participants should be done through family physicians and primary healthcare providers; also, we can utilize the public media to provide reliable resources and information concerning stroke. Future research should evaluate the effectiveness of educational programs and interventions in improving stroke awareness and assess their long-term impact on stroke outcomes. In addition, we recommend conducting more community-based studies about stroke, particularly in the rural areas of Saudi Arabia.

## Conclusions

This research study aimed to assess the awareness of acute stroke among the general population in the Western Region of Saudi Arabia. The findings indicate that the level of awareness of acute stroke among the general population in the Western Region of Saudi Arabia is moderate regarding acute stroke warning symptoms, risk factors, and appropriate stroke response among the population in this region.

These findings highlight the need for targeted educational interventions and awareness campaigns to improve knowledge about acute stroke in the Western Region of Saudi Arabia. Efforts should focus on providing accurate information about stroke symptoms, risk factors, and the importance of timely medical intervention. Strategies to enhance awareness should consider the preferences and accessibility of different age groups and diverse populations. By increasing awareness and knowledge about stroke, individuals will be better equipped to recognize the warning signs, seek appropriate medical attention promptly, and potentially reduce the burden and impact of stroke in the region.

## Appendices

The Questionnaire

Demographic data:

Gender:

◻ Male

◻ Female

Age:

◻<20

◻ 20-29 

◻ 30-39 

◻ 40-49

◻ 50-59   

◻ ≥60 

Nationality:

◻  Saudi

◻ Non-Saudi

Geographical Region:

◻ Makkah Region      

◻ Almadinah Region

◻ other

Residency:

◻ Urban Area       

◻ Rural Area 

Educational level:

◻ elementary   

◻ middle school    

◻ high school   

◻ above

Employment:

◻ Employed   

◻ Unemployed   

◻ Entrepreneur   

◻ Student   

◻ Retired

Occupational Field:

◻ Medical sector        

◻ Other 

Assessment of awareness about stroke:

Have you ever heard about stroke ? 

◻Yes

◻No

Have you or one of your family members ever had a stroke? 

◻Yes

◻No

Who is at high risk for stroke?

◻Children 

◻Young

◻Elderly 

What are the risk factors of stroke?

◻Obesity 

◻Smoking 

◻Hypertension 

◻Diabetes 

◻Heart diseases

What are the warning signs and symptoms of stroke?

◻Confusion

◻Weakness of the face or arm or leg

◻Sudden severe headache 

◻Slurred speech 

◻Vision problem

Is there any current treatment for stroke?

◻Yes

◻No

◻I don’t know 

What is the time frame to initiate thrombolysis in acute ischemic stroke ?

◻2 hours 

◻3 hours 

◻3.5 hours 

◻4.5 hours 

Can stroke cause death?

◻Yes

◻No

◻I don’t know 

Is stroke preventable?

◻Yes

◻No

◻I don’t know 

What is your source of information regarding stroke?

◻TV\Radio

◻ Internet

◻ School

◻Parents or Friends

◻Health campaign 

◻Health facilities
